# Construct validity and reliability of the Bilateral Vestibulopathy Questionnaire (BVQ)

**DOI:** 10.3389/fneur.2023.1221037

**Published:** 2023-11-01

**Authors:** Lisa van Stiphout, Jeremy Rolfes, Sophie Waardenburg, Merel Kimman, Nils Guinand, Angélica Pérez Fornos, Vincent Van Rompaey, Raymond van de Berg

**Affiliations:** ^1^Division of Balance Disorders, Department of Otorhinolaryngology and Head and Neck Surgery, School for Mental Health and Neuroscience, Maastricht University Medical Center, Maastricht, Netherlands; ^2^Department of Clinical Epidemiology and Medical Technology (KEMTA), Care and Public Health Research Institute (CAPHRI), Maastricht University Medical Centre, Maastricht, Netherlands; ^3^Service of Otorhinolaryngology Head and Neck Surgery, Department of Clinical Neurosciences, Geneva University Hospitals, Geneva, Switzerland; ^4^Department of Otorhinolaryngology and Head and Neck Surgery, Faculty of Medicine and Health Sciences, Antwerp University Hospital, University of Antwerp, Antwerp, Belgium

**Keywords:** bilateral vestibulopathy, Patient Reported Outcome Measure (PROM), questionnaire, vestibular impairment, symptoms, COSMIN

## Abstract

**Background:**

The Bilateral Vestibulopathy Questionnaire (BVQ) is a recently developed 54-item Patient Reported Outcome Measure (PROM) that evaluates the clinically important symptoms of bilateral vestibulopathy (BVP) and its impact on daily life. This study aimed to assess the construct validity and reliability of the BVQ in a large BVP cohort.

**Methods:**

Patients diagnosed with BVP were asked to complete a set of questionnaires, including the BVQ, the EuroQol-5D-5L, the Health Utilities Index, the Dizziness Handicap Inventory, the Hospital Anxiety and Depression Scale, and the Oscillopsia Severity Questionnaire. The construct validity of the BVQ was evaluated by confirmatory and exploratory factor analyses (CFA and EFA), followed by hypotheses testing and known groups validity. Structural properties were explored for each individual item. Reliability was assessed by testing the internal consistency of the BVQ constructs (Cronbach's alpha) and test–retest reliability [intraclass correlation coefficients (ICCs)].

**Results:**

A total of 148 patients with BVP (50% women, mean age 66 years) completed the set of questionnaires. The CFA did not show a satisfactory model in the original BVQ. However, the EFA showed a four-factor solution with 20 Likert-scale items related to oscillopsia, imbalance, emotion, and cognition. The succeeding CFA provided evidence for construct validity and an acceptable model of fit. Hypothesis testing confirmed that this shortened version validly measures the constructs to be measured. Statistically significant differences in scores between known groups were found, providing further support for good construct validity. The structural properties were acceptable. Cronbach's alpha confirmed good internal consistency for the four constructs, ranging from 0.80 to 0.89. The ICCs of the 20 Likert-scale items and four visual analog scale (VAS) items were interpreted as good (range 0.76–0.93).

**Conclusion:**

This study showed evidence of good construct validity of the new shortened version of the BVQ, consisting of four constructs with a total of 20 Likert-scale items and four VAS items. The final 24-item BVQ proved to be a reliable and valid multi-item PROM that captures the clinically important symptoms of BVP and evaluates its impact on daily life. Consequently, the BVQ enables the gathering of high-level evidence of treatment effectiveness in a systematic and quantitative manner.

## 1. Introduction

Bilateral vestibulopathy (BVP) is a heterogeneous and chronic disorder of both vestibular organs, and vestibular nerves, or the brain (1–3). It is characterized by a bilateral loss or reduction of vestibular function, which leads to symptoms in the physical, cognitive, and emotional domains (4). One of the main physical symptoms of BVP is unsteadiness when walking or standing, which worsens on uneven ground or in darkness (2). In addition, the majority of patients report movement-induced blurred vision (oscillopsia) (4). Cognitively, BVP is associated with difficulties when performing dual tasks, problems with concentration, forgetfulness, and impaired spatial orientation (4–8). Emotional symptoms related to BVP include sadness, anger, and (spatial) anxiety (3, 4). This broad spectrum of BVP symptoms can result in behavioral changes affecting daily activities and work since activities are either done more slowly and with increased attention or are completely avoided (4). Ultimately, these behavioral changes can result in diminished physical activity, reduced social functioning, deterioration of vitality, and even social isolation (4). Subsequently, the socioeconomic burden of BVP is substantial, as patients report an increased frequency of falls, increased healthcare utilization, and decreased productivity caused by symptom-related workplace absenteeism (9–12). Altogether, BVP negatively impacts the quality of life (13).

For diagnostic purposes, the Classification Committee of the Bárány Society established the BVP criteria, including the symptoms of unsteadiness and oscillopsia, together with a reduced vestibular function measured using vestibular reflex tests (2). However, to date, the severity and burden of the full spectrum of BVP symptoms from the patient's perspective cannot be evaluated in a validated and standardized manner. Currently, new treatment options that focus on alleviating BVP symptoms are in development. This increases the urgency for a validated assessment tool for the evaluation of the full spectrum of BVP symptoms, burden of disease, and impact on daily life to gather high-level evidence of treatment effectiveness (14–19). Therefore, the Bilateral Vestibulopathy Questionnaire (BVQ) was recently developed (20). The BVQ is a Patient Reported Outcome Measure (PROM), which includes 50 6-point Likert-scale items inquiring about imbalance, oscillopsia, cognitive symptoms, emotional symptoms, limitations and behavior, and social life, combined with four visual analog scale (VAS) items to inquire about limitations in daily life, perceived health, and expectations regarding future recovery. The content and format of the BVQ were based on a conceptual framework, a literature review, and individual semi-structured patient interviews, together with input from international experts. The development of the BVQ was in agreement with the COSMIN guideline for PROM development and proved to have good face and content validity, which was described extensively in a previous study (20). In summary, the BVQ was developed to capture the clinically important symptoms of BVP and evaluate its impact on daily life in order to assist in quantifying treatment efficacy and improve clinical decision-making.

As a next step for validating the BVQ, this study aimed to assess the psychometric properties of the BVQ by testing its construct validity and reliability in a large patient population.

## 2. Materials and methods

### 2.1. Study participants and data collection

This single-center study was performed at Maastricht University Medical Center+ over two periods of 3 months (period 1: April 2021–June 2021, period 2: November 2021–January 2022). Patients diagnosed with BVP according to the Bárány Society diagnostic criteria at the Department of Otorhinolaryngology and Head and Neck Surgery were asked to participate in this study. Inclusion criteria for BVP included imbalance and/or oscillopsia during walking or head movements, a reduced bithermal caloric response (sum of bithermal maximal peak slow phase velocity bilaterally <6°/s), and/or a reduced vestibular-ocular-reflex (VOR) gain as measured by the horizontal video Head Impulse Test (vHIT, bilateral VOR gain <0.6) and/or torsion swing test (VOR gain <0.1) (2). An extensive description of the test procedures was described previously (21, 22). It was required that patients be aged ≥18 years and able to understand the written Dutch language. Patients who had already participated in the development and content validity study of the BVQ were excluded from this study to avoid participant bias (20).

All patients were asked to complete the primary survey, including the BVQ, the 5-level EQ-5D questionnaire (EQ-5D-5L), the Health Utilities Index 3 (HUI-3), the Dizziness Handicap Inventory (DHI), the Hospital Anxiety and Depression Scale (HADS), and the Oscillopsia Severity Questionnaire (OSQ) (23–29). Additionally, patients were asked to fill out the retest BVQ three days after the primary survey.

Patients were preferably contacted by phone to participate in this study. If contact by phone was not possible, an invitation to participate was sent via e-mail or mail. After confirmation of participation, the online survey, which was built in Qualtrics (Qualtrics software, Version 20204, Qualtrics, Provo, UT, United States), was sent via e-mail. For patients who could not complete the survey online (e.g., no access to the Internet), a paper version was offered via postal services. After three days, patients received a second invitation to fill out the retest questionnaire (BVQ only) either via Qualtrics by automated e-mail or via postal services (paper version).

Demographic data such as date of birth and sex, as well as clinical data for BVP etiologies and vestibular test results (i.e., caloric test, torsion swing test, and horizontal vHIT), were collected from the electronic medical files provided by the Department of Otorhinolaryngology and Head and Neck Surgery at the Maastricht University Medical Center+. Vestibular test specifications as described by the Bárány Society were adhered (2).

### 2.2. Measures

#### 2.2.1. Bilateral Vestibulopathy Questionnaire

The Bilateral Vestibulopathy Questionnaire (BVQ) was developed to measure the full spectrum of BVP symptoms and their impact on daily life. After the development and analysis of the content validity, it consisted of seven constructs (imbalance, oscillopsia, other physical symptoms, cognition, emotion, behavior and limitations, and social life) with a total of 54 items (20).

A 6-point (Likert-type) scale was used for all seven constructs with the following anchor levels: always, frequently, regularly, sometimes, rarely, and never. In addition, the answer option “not applicable” was added to four questions in the construct “behavior and limitations.” Four items were answered using the VAS (0–100). The BVQ includes only level “B1” language, as defined in the Common European Framework of References for Languages. Documents of this level are supposed to be understood by 95% of the population speaking that language. The version of the BVQ after development and content validation is presented in Supplementary Table S1 (20).

For the purpose of psychometric assessment, mean construct scores were calculated by dividing the sum of all item scores by the number of items per construct. Item scores were categorized as: never 1, rarely 2, sometimes 3, regularly 4, frequently 5, and always 6. Items 23, 25, and 28 (construct emotion) were phrased positively and were, therefore, recoded inversely prior to analysis. The overall score of the BVQ was calculated as the sum of all average scores per construct, ranging from 7 to 42. The VAS items are presented per item, and an average for each item can be calculated for the total study population.

#### 2.2.2. EuroQol-5D-5L

The EQ-5D-5L is a validated multi-dimension questionnaire used to measure generic health status and health-related quality of life (HRQoL). It inquires about mobility, self-care, daily activities, pain/discomfort, anxiety/depression, and contains a visual analog scale for evaluation of the perceived health status. Each dimension contains five response levels (1 = no problems to 5 = unable to/severe problems), and reported answers can be converted into the EQ-5D-5L index score ranging from −0.059 to 1 (27, 29).

#### 2.2.3. Health Utilities Index 3

The HUI-3 is a validated multi-attribute health utility questionnaire used for the assessment of general health status and HRQoL (26). The questionnaire contains 15 items according to eight specific attributes: vision, hearing, speech, ambulation, dexterity, emotion, cognition, and pain, each with 5 or 6 levels for (dis)ability. Reported answers can be converted to HRQoL via a population-validated utility transformation function, yielding a score ranging from 1 (perfect health) to 0 (death). The HUI-3 global utility scores can be categorized into no disability (1.00), mild disability (0.89–0.99), moderate disability (0.70–0.88), and severe disability (<0.70) (26).

#### 2.2.4. Dizziness Handicap Inventory

The validated DHI is used for the assessment of the impact of dizziness on quality of life. It consists of 25 items evaluating three domains (physical, emotional, and functional) with three answer options (“yes” 4 points, “sometimes” 2 points, and “no” 0 points). The total score ranges from 0 (“no difficulty”) to 100 (“maximum difficulty”), which provides information about the self-perceived handicap. Scores between 16 and 34 indicate mild handicaps; scores between 36 and 52 indicate moderate handicaps; and scores equal to or higher than 54 indicate severe handicaps (24).

#### 2.2.5. Hospital Anxiety and Depression Scale

The HADS is a validated questionnaire used to identify possible or probable anxiety and/or depression through self-assessment. It consists of two subscales (anxiety and depression) with seven items each. Item scores range from 0 to 3, and for each subscale, the scores are summed. A score between 0 and 7 points indicates no present anxiety or depression; a score between 8 and 10 points indicates possible anxiety or depression; and scores exceeding 10 points indicate probable anxiety or depression (23, 25).

#### 2.2.6. Oscillopsia Severity Questionnaire

The OSQ is a 9-item instrument investigating oscillopsia severity in different daily life situations. A 5-point (Likert-type) scale is used: 1 (never), 2 (seldom), 3 (sometimes), 4 (often), and 5 (always). The sum of all item scores is averaged (range 1–5), and a mean score of 3 or higher indicates moderate to extreme oscillopsia severity (28). The internal consistency of the OSQ was previously tested and was considered good (28).

### 2.3. Data analysis

**Figure 1:**
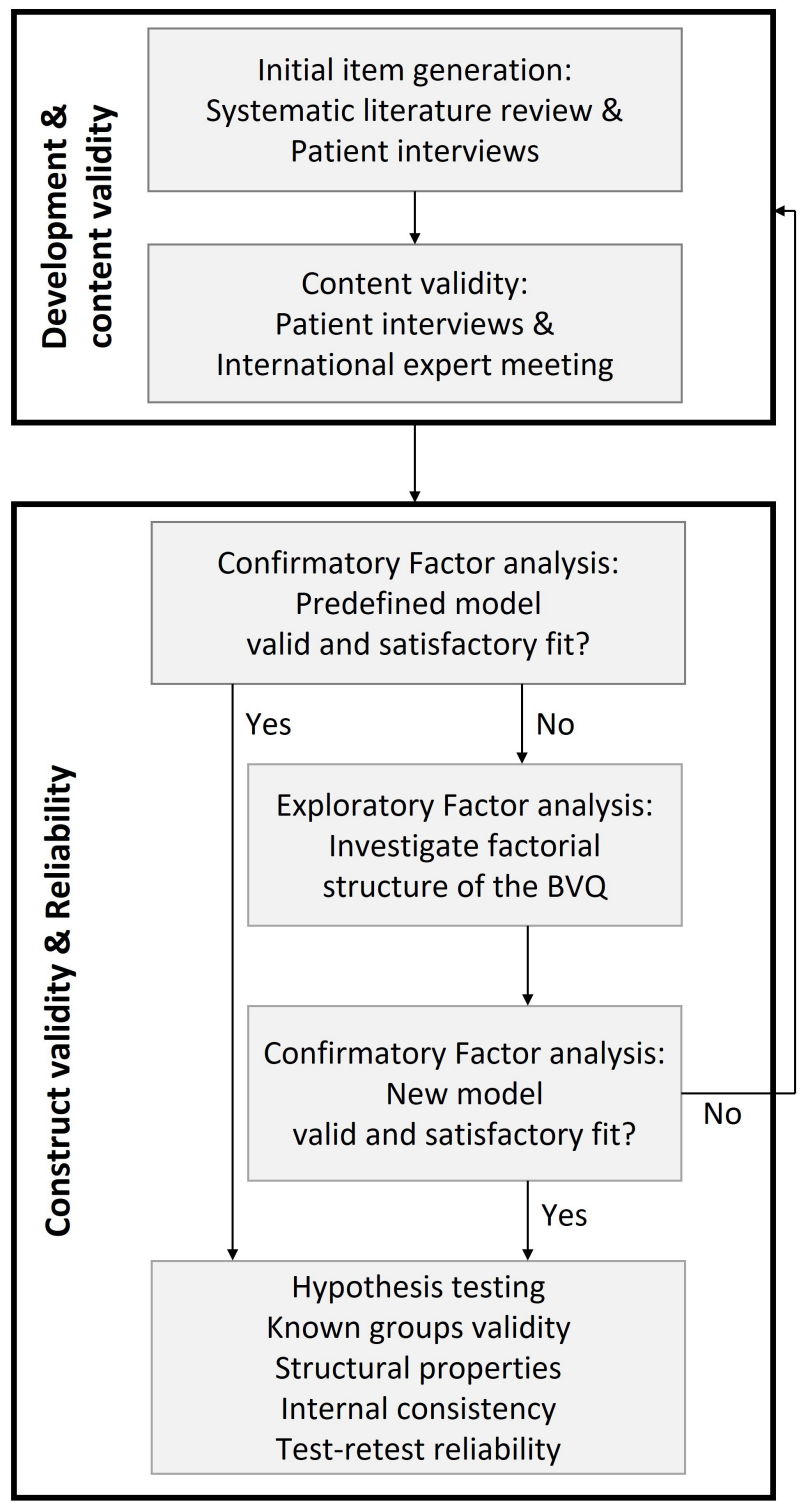
Flowchart summarizing all steps of BVQ development and validation. The development and content validity phases (first box) were described extensively in a previous study (20).

Following the development and content validity of the 54-item questionnaire [published previously by van Stiphout et al. (20)], the first step involved a confirmatory factor analysis (CFA) to test whether the data fit the predefined factor structure of the BVQ. In case the CFA failed to show a valid and satisfactory model fit, an exploratory factor analysis (EFA) was performed to investigate the factorial structure of the BVQ and to screen for potential discrepancies with the original BVQ constructs. Subsequently, a CFA was again performed on the new model to confirm the construct validity, followed by hypotheses testing, known groups validity, investigation of structural properties, internal consistency, and test–retest reliability. All steps described above are represented in a flowchart in Figure 1. IBM SPSS Statistics version 28 and R Studio 2021 were used for data analysis. The occurrence of missing values was prevented by installing the requirement of filling in only one answer per item and by the necessity to answer each item before being able to proceed with the survey via the online survey tool Qualtrics. These instructions were also written down on the paper version and emphasized by phone for the patients who participated via postal services.

#### 2.3.1. Construct validity

##### 2.3.1.1. Confirmatory and exploratory factor analyses

To test whether the data fit the predefined factor structure, a CFA with robust maximum likelihood estimation was performed. Goodness-of-fit indices were assessed to evaluate the model's fit to the data. This included the chi-square index, comparative fit index (CFI), Tucker–Lewis index (TLI), the root mean square error of approximation (RMSEA), and the standardized root mean square residual (SRMR). A “good fit” was represented by an insignificant chi-square index, CFI, and TLI values exceeding 0.90, and SRMR and RMSEA values below 0.08 (30, 31). Convergent validity was evaluated by the size of the factor loadings and the average variance extracted (AVE) for each factor. An AVE above 0.5 was acceptable, and an AVE exceeding 0.7 was considered very good. Discriminant validity, indicating whether each construct has enough discriminant validity from the other constructs, was considered good when the correlation coefficients between the constructs were below 0.8 (32).

In case the model fit was not satisfactory, the factorial structure of the BVQ (excluding the four VAS items) was analyzed by an EFA. Then, the Kaiser–Meyer–Olkin (KMO) measure of sampling adequacy was calculated and was considered “good” when exceeding 0.80 (33). In addition, Bartlett's test of sphericity was used to investigate the correlation structure and was considered “good” when significant (*p* < 0.05) (33). The scree plot was assessed to indicate the number of constructs with eigenvalues >1. The exploratory factor analysis was performed with oblique rotation (direct oblimin) using principal component analysis, with a cutoff point for item loadings only larger than 0.60.

##### 2.3.1.2. Hypothesis testing

The construct validity was further evaluated by testing *a priori* established hypotheses about expected associations between the BVQ and other PROMs.

1. Overall, it was hypothesized that the total BVQ score had a moderate negative correlation with HRQoL. This would imply that higher BVQ scores are associated with lower HRQoL measured with the EQ-5D-5L index score and the HUI-3 overall utility score (13).
2. Next, it was hypothesized that a higher total BVQ score was moderately to strongly and positively correlated with the self-perceived dizziness-related handicap as measured by the sum of all constructs of the DHI (4).
3. More specifically, it was hypothesized that a higher score in the BVQ construct emotion was moderately and positively correlated with a higher possibility of having anxiety or depression (i.e., higher scores in the HADS Anxiety and/or Depression constructs) (4). In line with this, it was expected that higher scores in the BVQ construct emotion were also moderately and positively correlated to higher scores in the emotional domain of the DHI and moderately and negatively correlated with the HUI-3 emotion score (4).
4. A moderate and negative correlation was expected between the BVQ construct cognition and the HUI-3 cognition score (4).
5. Regarding the physical symptoms, it was hypothesized that there was a weak and negative correlation between the BVQ imbalance construct and the HUI-3 ambulation score and a weak and positive correlation with the physical domain of the DHI since both the HUI-3 and the DHI inquire about difficulties while walking without specifying imbalance.
6. Finally, it was expected that higher scores in the BVQ oscillopsia construct were strongly and positively correlated with higher average scores of the OSQ since it was hypothesized that both the oscillopsia construct of the BVQ and the OSQ measure the same concept (20, 28). On the contrary, it was expected that there was a weak negative correlation between the BVQ oscillopsia construct and the HUI-3 vision score since the HUI-3 vision attribute measures vision in static conditions, whereas the BVQ oscillopsia construct measures vision in dynamic conditions. The latter being one of the main symptoms of BVP.

The hypotheses and strength of the associations were tested with the Pearson correlation or Spearman's rank-order correlation coefficient, depending on whether the data were normally distributed or not. The strength of the correlations was considered “very strong” if the correlation coefficient was >0.90 and “strong” if the correlation coefficient was between 0.70 and 0.90. The strength of the correlation was considered “moderate” and “weak” if the correlation coefficient was between 0.50–0.70 and 0.30–0.50, respectively. A correlation with a correlation coefficient <0.30 was assumed to be very weak or absent. A *p*-value below 0.05 was considered significant (34).

##### 2.3.1.3. Known groups validity

Known groups validity was tested by comparing mean scores between groups that, in theory, should have different scores. Four groups were differentiated based on the classification of the DHI into a no handicap group (0–15 points), a mild handicap group (16–34 points), a moderate handicap group (36–52 points), and a severe handicap group (54+ points) (24). Before analysis, it was expected that patients with severe perceived dizziness-related handicaps as measured by the DHI would have a higher total BVQ score compared with the total BVQ scores of patients with no or mild perceived dizziness-related handicaps. This was expected to result in a significant difference in BVQ scores between the four DHI severity groups. Scores of known groups were compared using a one-way analysis of variance (ANOVA) with a Bonferroni *post-hoc* test. A *p*-value <0.05 was considered significant.

#### 2.3.2. Structural properties

Score distributions were presented graphically with stacked bar charts for each BVQ item. When at least 15% of the respondents scored the lowest or highest possible score, a floor or ceiling effect was considered present (35).

#### 2.3.3. Reliability

##### 2.3.3.1. Internal consistency

Cronbach's alpha was calculated separately for each construct of the BVQ to measure the internal consistency of each item in its respective construct. A low Cronbach's alpha suggested a low or absent correlation between each item in the same construct, whereas a very high Cronbach's alpha indicated that the items measured identical concepts. Therefore, Cronbach's alpha was considered good when it ranged from 0.70 to 0.95 (36).

##### 2.3.3.2. Test–retest reliability

During testing period 2, patients were asked to complete the BVQ a second time, three days after the initial completion of the first survey (test–retest). The intraclass correlation coefficient (ICC) was calculated for each item of the BVQ to test the reliability between the two measurements (59). The ICC and 95% confidence interval were calculated based on absolute agreement and a two-way mixed-effects model. The reliability was considered “good” if the ICC was at least 0.70 (36).

### 2.4. Ethical considerations

This study was conducted in accordance with the legislation and ethical standards on human experimentation in the Netherlands and in accordance with the Declaration of Helsinki (amended version 2013). The medical ethical committee of Maastricht UMC+ approved this study (METC 2020-2215), and written informed consent was obtained from all patients participating in this study.

## 3. Results

### 3.1. Patient characteristics

A total of 148 patients diagnosed with BVP [50% women, mean age 66 years (range 20–89 years)] completed the set of questionnaires. In all, 91% participated online, and 9% completed the paper version. In 37.2% of the cases, the BVP etiology was idiopathic. Other etiologies were genetic (17.6%), ototoxicity (13.5%), infection (11.5%), Menière's disease (5.4%), auto-immune (5.4%), vestibular migraine (2.7%), neurodegenerative (2.0%), mixed etiologies (2.0%), congenital (1.4%), iatrogenic (0.7%), and vascular (0.7%). Available data on vestibular reflex tests for the participating patients with BVP showed a mean bithermal caloric test result of 1.9°/s and 1.8°/s for the right and left ear, respectively (i.e., the sum of the bithermal maximal peak slow phase velocity bilaterally). The mean horizontal vHIT VOR gain was 0.32 and 0.33 for right and left, respectively, and the mean VOR gain for the torsion swing test was 0.13.

### 3.2. Construct validity

#### 3.2.1. Confirmatory and exploratory factor analyses

The CFA applied to the predefined model from the development and content validity phases failed to show a satisfactory model fit. Subsequently, all 50 Likert-type items of the predefined model were assessed in an EFA with oblique rotation (direct oblimin). The KMO was 0.86, and therefore the sampling adequacy for the analysis was considered good. In addition, the correlation structure was considered good since Barlett's test of sphericity was significant [$\chi_{\left(210\right)}^{2}$ = 1,777, *p* < 0.001]. The scree plot indicated that four factors would be suitable for the 50 Likert-type items (Supplementary Figure S1). The EFA with a cutoff point for item loadings larger than 0.60 resulted in a solution of 20 items, which accounted for 65% of the variance (Table 1 and Supplementary Table S2). Details regarding deleted items and statistical results from the EFA are presented in Supplementary Tables S2–S4.

**Table 1:** Constructs, items, and response options of the Bilateral Vestibulopathy Questionnaire after exploratory factor analysis.

**Bilateral vestibulopathy questionnaire**		
**Construct/item**		**Answer scale**
**Oscillopsia**		
1	I have blurred vision while walking.	*1 (never)−6 (always)*
2	I have blurred vision while traveling (such as being on a train, bus, car, or on a bike).	*1 (never)−6 (always)*
3	When walking, I have to stand still to recognize faces or to read (road) signs.	*1 (never)−6 (always)*
4	I have blurred vision while chewing on my food.	*1 (never)−6 (always)*
5	I have blurred vision when quickly turning my head.	*1 (never)−6 (always)*
6	I experience difficulties with fast head movements, like turning my head to the right or left when crossing the street.	*1 (never)−6 (always)*
**Imbalance**		
7	I experience imbalance during daily activities.	*1 (never)−6 (always)*
8	I experience imbalance when walking on uneven surfaces (like in the woods, at the beach, or in the snow).	*1 (never)−6 (always)*
9	I experience imbalance when walking in reduced light.	*1 (never)−6 (always)*
10	When walking, I need to pay attention to the ground to avoid falling.	*1 (never)−6 (always)*
11	I experience imbalance while changing positions (such as crouching, bending down, reaching, or standing up).	*1 (never)−6 (always)*
12	I must pay close attention to my balance.	*1 (never)−6 (always)*
**Emotion**		
13	I feel lonely.	*1 (never)−6 (always)*
14	I feel I have control over my life.	*1 (never)−6 (always)*
15	I don't feel confident when performing daily activities.	*1 (never)−6 (always)*
16	I feel sad.	*1 (never)−6 (always)*
17	I am embarrassed by my balance problems.	*1 (never)−6 (always)*
**Cognition**		
18	I am forgetful.	*1 (never)−6 (always)*
19	I find it difficult to concentrate.	*1 (never)−6 (always)*
20	I experience difficulties with doing more than one thing at a time.	*1 (never)−6 (always)*

The items were developed in Dutch for the Dutch population. The questionnaire was translated into English only for the purpose of this publication.

Finally, a CFA was performed on the new, shortened model of the BVQ. Overall, the goodness-of-fit indices suggested an acceptable model with a satisfactory fit. Although the chi-square statistic was significant [$\chi_{\left(164,\;\text{N =}\;148\right)}^{2}$ = 292.83, *p* = 0.00], a good fit was represented by the CFI (0.92), the TLI (0.91), the SRMR (0.06), and the RMSEA (0.07). The convergent validity was considered good, with moderate to large sizes of all factor loadings and values for the AVE for each construct exceeding 0.7. The correlation coefficients between the constructs ranged from 0.27 to 0.61, which were all below 0.80, supporting discriminant validity between the constructs. The visualization of the structural equation of the 20-item model is shown in Supplementary Figure S2.

#### 3.2.2. Hypothesis testing

As hypothesized, there was a significant moderate negative correlation between the total BVQ score and HRQoL as measured with the EQ-5D-5L index score (Spearman's rho = −0.54, *p* < 0.001) and the HUI-3 overall utility score (Pearson's r = −0.52, *p* < 0.001). Furthermore, total BVQ scores were strongly positively correlated with the self-perceived dizziness-related handicap as measured by the sum of all constructs of the DHI (Pearson's r = 0.78, *p* < 0.001).

When considering specific constructs, the BVQ construct emotion was moderately correlated with the possibility of having anxiety or depression according to the HADS Anxiety and Depression constructs (Spearman's rho = 0.69 and 0.69, *p* < 0.001 respectively). In line with this, the BVQ construct emotion was strongly positively correlated with the emotional domain of the DHI (Spearman's rho = 0.72, *p* < 0.001) and moderately negatively correlated with the HUI-3 emotion score (Spearman's rho = −0.57, *p* < 0.001). In addition, a moderate and negative correlation was found between the BVQ construct cognition and the HUI-3 cognition score (Spearman's rho = −0.66, *p* < 0.001). Regarding the physical symptoms, a borderline weak negative correlation was found between the BVQ imbalance construct and the HUI-3 ambulation score (Spearman's rho = −0.50, *p* < 0.001) and a weak positive correlation with the physical domain of the DHI (Spearman's rho = 0.39, *p* < 0.001). Finally, scores in the BVQ oscillopsia construct were strongly and positively correlated with the OSQ (Spearman's rho = 0.78, *p* < 0.001) and weakly correlated with the HUI-3 vision attribute utility score (Spearman's rho = −0.39, *p* < 0.001).

#### 3.2.3. Known groups validity

**Figure 2:**
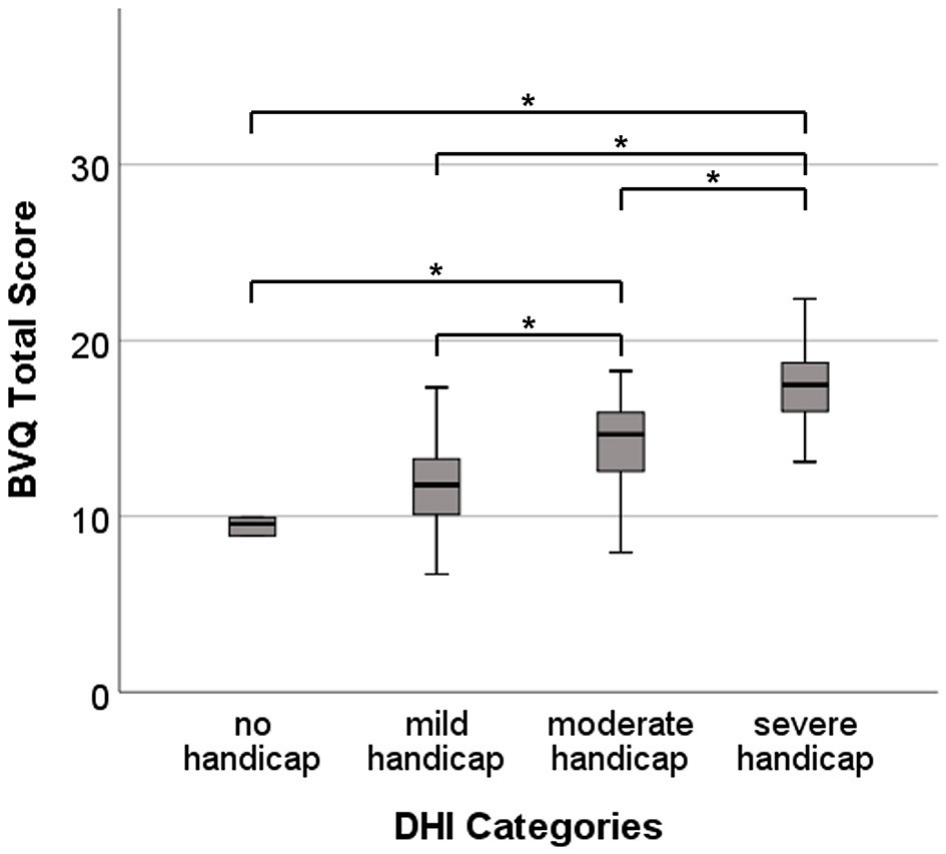
Total BVQ scores for the four self-perceived dizziness-related handicap groups (no handicap, mild handicap, moderate handicap, and severe handicap) according to the Dizziness Handicap Inventory (DHI) total score classification. Each box plot represents 25–75 percentiles, bold black lines indicate the median, and asterisks (*) illustrate statistically significant differences.

A statistically significant difference in total BVQ score was found between the DHI severity groups as determined by one-way ANOVA [F_(3)_ = 49.01, *p* < 0.001]. The Bonferroni *post-hoc* test revealed that the total BVQ score was significantly different between all groups, except between the mild and no handicap groups (Figure 2 and Supplementary Tables S5, S6).

### 3.3. Structural properties

**Figure 3:**
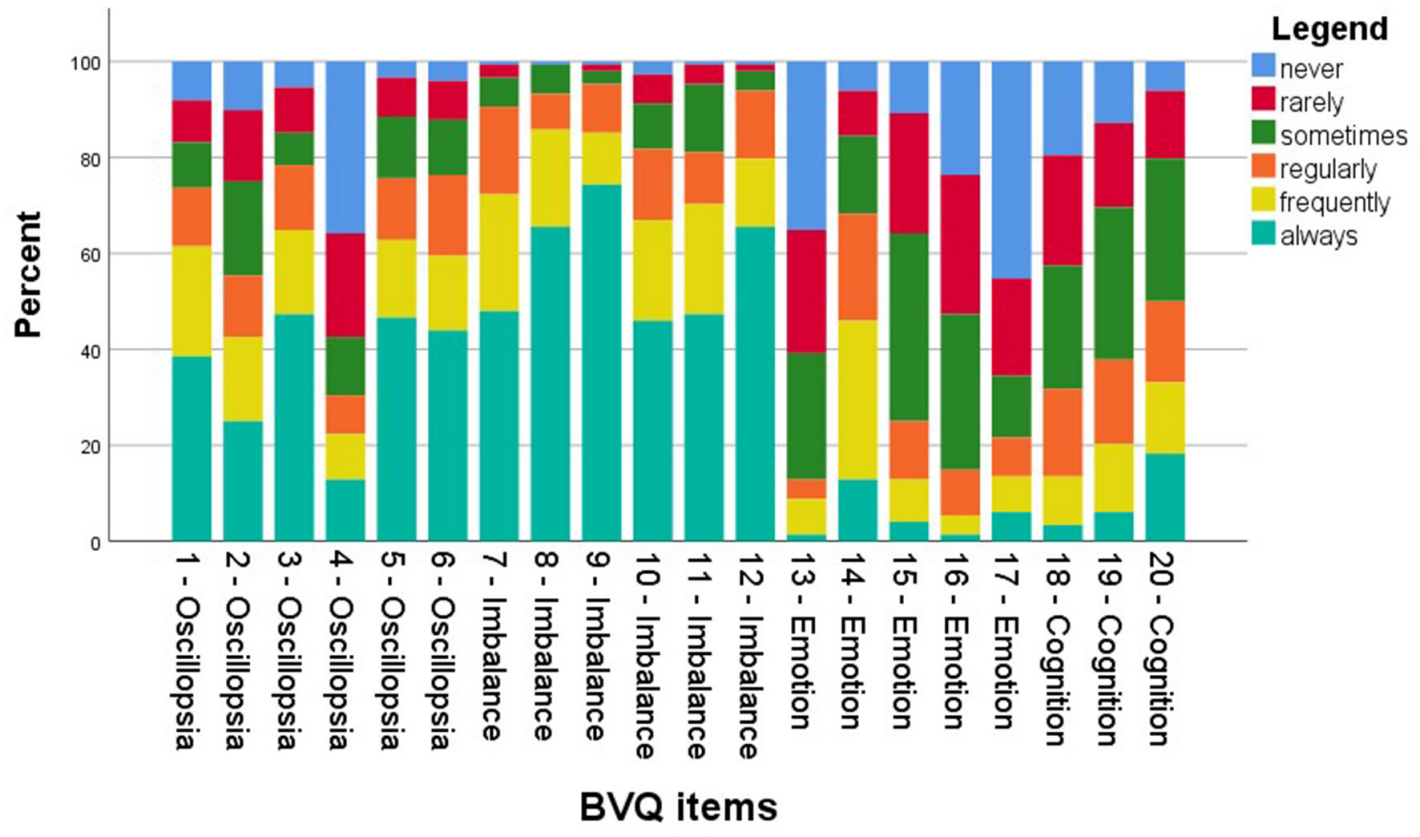
Stacked bar chart of response distribution of the Bilateral Vestibulopathy Questionnaire (BVQ).

Analysis of structural properties demonstrated that all items, except for item 8, included responses across the full range of response options (Figure 3). There were no missing values. A floor effect was found in items 4 (oscillopsia), 13 (emotion), 16 (emotion), 17 (emotion), and 18 (cognition). Ceiling effects were seen in almost all oscillopsia items (except for item 4), all imbalance items, and item 20 (cognition).

### 3.4. Reliability

#### 3.4.1. Internal consistency

All constructs showed Cronbach's alpha within the range of 0.70 to 0.95, providing evidence for internally consistent (homogeneous) scales. Constructs with their respective Cronbach's alpha are presented in Table 2.

**Table 2:** Internal consistency of the Bilateral Vestibulopathy Questionnaire (BVQ), as assessed by calculating Cronbach's alpha^a^ for each construct separately.

**Construct**	**Cronbach's alpha^a^**
Oscillopsia	0.89
Imbalance	0.88
Emotion	0.80
Cognition	0.89

^a^Internal consistency was considered good when Cronbach's alpha was between 0.70 and 0.95.

#### 3.4.2. Test–retest reliability

In all, 88% of the patients [mean age 65 years (range 27–87 years), 46% women] completed the test–retest measurement. The mean time between the test and retest was 4 ± 1.5 days. The ICCs for the single Likert-scale and VAS items were good, and all fell above the recommended cutoff value of at least 0.70 (Table 3). The mean scores and standard deviation of single items were nearly identical with little variation.

**Table 3:** Intraclass correlation coefficient (ICC) with 95% confidence intervals (CI) of the Bilateral Vestibulopathy Questionnaire (BVQ) test (T1) and retest (T2) (*N* = 87), with means and standard deviations (SD).

**Item**	**T1**		**T2**		
	**Mean**	**SD**	**Mean**	**SD**	**ICC**	**95% CI**
**Oscillopsia**						
1	4.54	1.63	4.54	1.54	0.88	0.82–0.92
2	3.87	1.63	3.71	1.61	0.83	0.74–0.89
3	4.84	1.63	4.77	1.55	0.89	0.83–0.93
4	2.60	1.62	2.59	1.58	0.81	0.72–0.88
5	4.68	1.47	4.31	1.71	0.82	0.72–0.89
6	4.55	1.53	4.32	1.60	0.87	0.82–0.93
**Imbalance**						
7	5.00	1.12	4.84	1.18	0.87	0.80–0.91
8	5.40	0.99	5.37	1.15	0.86	0.78–0.91
9	5.52	1.03	5.31	1.25	0.87	0.80–0.92
10	4.99	1.36	4.78	1.36	0.91	0.86–0.94
11	4.86	1.32	4.70	1.40	0.82	0.73–0.88
12	5.38	1.07	5.14	1.20	0.88	0.81–0.93
**Emotion**						
13	2.23	1.31	2.13	1.21	0.87	0.80–0.92
14	2.89	1.32	2.84	1.26	0.80	0.70–0.87
15	3.01	1.22	3.08	1.29	0.79	0.68–0.87
16	2.43	1.15	2.44	1.21	0.89	0.83–0.93
17	2.31	1.53	2.38	1.45	0.87	0.80–0.92
**Cognition**						
18	2.74	1.28	2.78	1.22	0.93	0.89–0.95
19	2.99	1.35	3.00	1.33	0.87	0.80–0.92
20	3.55	1.45	3.49	1.49	0.87	0.80–0.92
**Visual analog scale items**						
21	58.14	25.59	55.52	25.36	0.87	0.81–0.92
22	71.30	24.08	70.28	22.94	0.76	0.64–0.85
23	25.71	27.59	28.77	29.61	0.92	0.88–0.95
24	63.39	23.16	65.14	22.04	0.80	0.70–0.87

ICC estimates were calculated based on absolute agreement and two-way mixed-effects models. Following recommendations by Terwee et al., the reliability was positively rated when the ICC was at least 0.70.

## 4. Discussion

This study demonstrated that the 24-item BVQ is a valid and reliable PROM to assess the spectrum of BVP symptoms and their impact on daily life. Its development and validation were strengthened through substantial patient input at different stages of the process, closely following the COSMIN guidelines (37, 38). Moreover, high-level evidence of good construct validity was provided by the CFA, hypotheses testing, and known groups validity, and good reliability was reflected by satisfactory internal consistency and high test–retest reliability. The major advantage of the BVQ is that it is the first validated assessment tool to gather evidence of treatment effectiveness on the full spectrum of BVP symptoms, burden of disease, and impact on daily life.

After extensive psychometric testing, the final version of the BVQ, which includes 20 Likert-type items and four VAS items, is considerably shorter compared to the version after the development and content validity phases (20). This is beneficial, as a shorter questionnaire eases its use for the patient and clinician. Although many items from the development and content validity phases are not included, it is important to note that the items specifically added at the patients' request are all still included in the final shorter version. The previous constructs “Limitations and Behavior” and “Social Life” are not included in the final version, yet both constructs are represented in the VAS items and construct emotion, respectively. In summary, even though the final version is considerably shorter, all original concepts from the theoretical framework are still represented (20).

Overall, the predefined hypotheses regarding other well-known and validated PROMs were confirmed. As expected, the BVQ had a moderate correlation with generic measures such as the EQ-5D-5L and the HUI-3. Although a strong correlation between HRQoL and BVP is expected, the correlation between the BVQ and generic measures developed for measuring HRQoL (EQ-5D-5L and HUI-3) was only moderate since these generic questionnaires are not able to capture BVP symptoms accurately (4). Compared to generic measures, the BVQ inquires about specific aspects of BVP relevant to the patient, whereas the EQ-5D-5L and the HUI-3 include domains such as pain and speech, which are not fully applicable to the BVP patient population. The BVQ is therefore expected to be more sensitive to disease-specific changes, which is necessary for measuring therapy effectiveness. When considering total scores, the DHI was found to be strongly correlated with the BVQ. A notable finding, however, is the difference in the strength of correlation of the DHI subscores of the emotional and physical domains with the respective BVQ constructs. The emotional domain of the DHI proved to be strongly correlated with the emotional construct of the BVQ, whereas the physical domain of the DHI was only weakly correlated with the BVQ construct imbalance. This is interesting since some of the physical items in the DHI also inquire about difficulties while walking. Nevertheless, not all items from the DHI psychical domain are related to difficulties while walking (e.g., “Does turning over in bed increase your problem?”), and the items that are related to difficulties while walking and imbalance are not as specific as the imbalance items in the BVQ (e.g., in the DHI, “Does walking down a sidewalk increase your problem?”). This underlines the importance of measuring imbalance within its own construct with specific items related to BVP. Finally, the weak correlation between the BVQ oscillopsia construct and the HUI-3 vision score emphasizes the importance of inquiring about vision in dynamic conditions (oscillopsia). In the HUI-3, dynamic conditions are not considered, and therefore oscillopsia, one of the main symptoms of BVP, is not adequately captured. This again illustrates the relevance of a disease-specific measure.

The structural properties of the BVQ were satisfactory, with responses across the full range of response options and no missing values. Ceiling effects were seen in almost all oscillopsia and imbalance items, reflecting the severity of the two main BVP symptoms as described in the literature (2, 4). These ceiling effects are expected to be less pronounced after therapeutic interventions focusing on (partial) recovery of the vestibulo-ocular and vestibulo-collic reflexes, indicating symptom improvement (therapy effectiveness). In future studies examining responsiveness and treatment effectiveness, it is essential to investigate whether minor symptom improvements, detectable through objective clinical measures, also manifest in the relevant aspects of the BVQ.

The good reliability of the BVQ was reflected by satisfactory internal consistency and high test–retest reliability. Good internal consistency of the constructs indicated that the items were sufficiently homogeneous to be pooled within the constructs. In addition, the BVQ was shown to yield consistent results, as reflected by the high test–retest reliability measured by the ICC.

It is important to note that in this study, mean construct scores and a total BVQ score (sum of mean construct scores) were used without conducting an official weighted scoring study first (i.e., Do all construct scores contribute equally to a total score?). However, the psychometric analyses performed in this study were not impacted by the scoring protocol, except for the hypothesis testing. Nevertheless, a total BVQ score based on the sum of the mean scores of each construct is currently not yet recommended to be used in effectiveness studies. Mean construct scores, on the other hand, can already be used in future studies. One of the assets of this study is that the psychometric tests were not applied to the same population as those involved in the development and content validity phases. This contributes to reducing participant bias. Finally, the BVQ development and validation were strengthened through substantial patient input at different stages of the process, closely following the COSMIN guidelines, and by the significant contribution of an international team of clinical experts, which will help cross-cultural validation and implementation (37, 38).

Future work on the BVQ includes an official weighted scoring study and translation into different languages, followed by cross-cultural validation to facilitate its use internationally (37). Furthermore, it may also be of added value to explore the BVQ in a unilateral vestibulopathy population. This could help determine whether the BVQ can also distinguish between symptoms of unilateral and bilateral vestibulopathy.

## 5. Conclusion

The 24-item BVQ is a valid and reliable PROM to assess the spectrum of BVP symptoms and their impact on daily life. The major asset of the BVQ is that it captures all patient-relevant aspects of BVP and is, therefore, able to provide high-level evidence of treatment effectiveness in a systematic and quantitative manner.

## Data availability statement

The raw data supporting the conclusions of this article will be made available by the authors, without undue reservation.

## Ethics statement

The studies involving humans were approved by the Medical Ethical Committee of Maastricht UMC+ (METC 2020-2215). The studies were conducted in accordance with the local legislation and institutional requirements. The participants provided their written informed consent to participate in this study.

## Author contributions

LS, MK, and RB designed the study. LS and JR ensured data acquisition. LS, JR, and SW conducted the analysis. In addition to the academic and clinical background of SW, she is trained in the skills necessary to perform robust statistical PROM analyses. LS wrote the manuscript. MK and RB supervised the writing. SW, JR, NG, AP, and VV reviewed the manuscript. All authors contributed to the article and approved the submitted version.
