# Supplementary material for: Construct validity and reliability of the Bilateral Vestibulopathy Questionnaire (BVQ)
Source: Front Neurol. 2023 Nov 1;14:1221037. doi: 10.3389/fneur.2023.1221037 (PMC10646559; doi:10.3389/fneur.2023.1221037)
Supplement: Supplementary file 1 [file Data_Sheet_1.pdf]

Supplementary Materials

Construct Validity and Reliability of the Bilateral Vestibulopathy Questionnaire (BVQ)

Supplementary Table 1 Constructs, items, and response options of the Bilateral Vestibulopathy Questionnaire after the development and content validity phase (from van Stiphout et al. 2022 (1)). The items were developed in Dutch for the Dutch population. These items were translated into English for the purpose of this publication.

	**Bilateral Vestibulopathy Questionnaire**	
	**Construct/Item**	**Answer scale**
	Imbalance	
1	I experience imbalance during daily activities.	*1 (never) – 6 (always)*
2	I experience imbalance when walking on uneven surfaces (like in the woods, at the beach or in the snow).	*1 (never) – 6 (always)*
3	I experience imbalance when walking in reduced light.	*1 (never) – 6 (always)*
4	When walking, I need to pay attention to the ground to avoid falling.	*1 (never) – 6 (always)*
5	I have fallen.	*1 (never) – 6 (always)*
6	I have come close to falling.	*1 (never) – 6 (always)*
7	I experience imbalance while changing positions (such as crouching, bending down, reaching or standing up).	*1 (never) – 6 (always)*
	Oscillopsia	
8	I have blurred vision while walking.	*1 (never) – 6 (always)*
9	I have blurred vision while travelling (such as being on a train, bus, car or on a bike).	*1 (never) – 6 (always)*
10	When walking, I have to stand still to recognize faces or to read (road) signs.	*1 (never) – 6 (always)*
11	I have blurred vision while chewing on my food.	*1 (never) – 6 (always)*
12	I have blurred vision when quickly turning my head.	*1 (never) – 6 (always)*
	Other psychical symptoms	
13	I experience difficulties with fast head movements, like turning my head to the right or left when crossing the street.	*1 (never) – 6 (always)*
14	I experience light-headedness when standing up fast.	*1 (never) – 6 (always)*
15	I feel tired.	*1 (never) – 6 (always)*
	Cognition	
16	I must pay close attention to my balance.	*1 (never) – 6 (always)*
17	I easily get lost in new places.	*1 (never) – 6 (always)*
18	I find it difficult to judge distances.	*1 (never) – 6 (always)*
19	I am forgetful.	*1 (never) – 6 (always)*
20	I find it difficult to concentrate.	*1 (never) – 6 (always)*
21	I experience difficulties with doing more than one thing at a time.	*1 (never) – 6 (always)*
22	I experience difficulties with doing things while walking.	*1 (never) – 6 (always)*
	Emotion	
23	I feel happy.	*1 (never) – 6 (always)*
24	I worry a lot.	*1 (never) – 6 (always)*
25	When performing daily activities, I feel confident.	*1 (never) – 6 (always)*
26	I feel lonely.	*1 (never) – 6 (always)*
27	I am afraid to fall.	*1 (never) – 6 (always)*
28	I feel I have control over my life.	*1 (never) – 6 (always)*
29	I am bothered by fast movements surrounding me.	*1 (never) – 6 (always)*
30	I am bothered by noisy environments.	*1 (never) – 6 (always)*
31	I am bothered by busy environments surrounding me.	*1 (never) – 6 (always)*
32	I don't feel confident when performing daily activities.	*1 (never) – 6 (always)*
33	I feel sad.	*1 (never) – 6 (always)*
34	I am embarrassed by my balance problems.	*1 (never) – 6 (always)*

	Behavior and limitations	
35	I feel limited in daily life activities.	*1 (never) – 6 (always)*
36	I avoid daily life activities.	*1 (never) – 6 (always)*
37	I feel limited when performing vigorous activities.	*1 (never) – 6 (always) – N/A*
38	I avoid performing vigorous activities.	*1 (never) – 6 (always) – N/A*
39	I feel limited when performing moderate activities.	*1 (never) – 6 (always)*
40	I avoid performing moderate activities.	*1 (never) – 6 (always)*
41	I feel limited in travelling.	*1 (never) – 6 (always)*
42	I avoid travelling.	*1 (never) – 6 (always)*
43	I feel limited when driving a car.	*1 (never) – 6 (always) – N/A*
44	I avoid driving a car.	*1 (never) – 6 (always) – N/A*
45	I feel limited in my social activities.	*1 (never) – 6 (always)*
46	I avoid social activities.	*1 (never) – 6 (always)*
47	I need to perform daily activities slower.	*1 (never) – 6 (always)*
	Social life	
48	My symptoms negatively affect my close relationships.	*1 (never) – 6 (always)*
49	People don't understand my problem.	*1 (never) – 6 (always)*
50	I need the help of other people to perform daily activities.	*1 (never) – 6 (always)*
	VAS scale questions	
51	How limited do you feel in daily life?<br>The scale below is numbered from 0 to 100.<br>0 means that you are not limited in daily life.<br>100 means that you are extremely limited in daily life.<br>Mark an X on the scale to indicate how much you feel limited in daily life.	*Scale 0 (not limited in daily life) – 100 (extremely limited in daily life)*
52a	What is the most important problem you would like to have improved?<br>Please fill in your answer here:	*Open answer*
52b	How much does this problem affect your life (see the answer you filled in at question 52a)?<br>The scale below is numbered from 0 to 100.<br>0 means the problem does not affect your life.<br>100 means the problem affects your life extremely.<br>Mark an X on the scale to indicate how much you suffer from this symptom.	*Scale 0 (no suffering) – 100 (extreme suffering)*
53	How hopeful are you that you will get better?<br>The scale below is numbered from 0 to 100.<br>0 means you are not hopeful that you will get better.<br>100 means you are extremely hopeful that you will get better.<br>Mark an X on the scale to indicate how hopeful you are that you will get better.	*Scale 0 (not hopeful) – 100 (extremely hopeful)*
54	How would you rate your health today?<br>The scale below is numbered from 0 to 100.<br>0 means your health is extremely bad.<br>100 means your health is extremely good.<br>Please note: the scale below is marked from 'extremely bad' (0) to 'extremely good' (100).<br>Mark an X on the scale to indicate how you feel today.	*Scale 0 (extremely bad) – 100 (extremely good)*

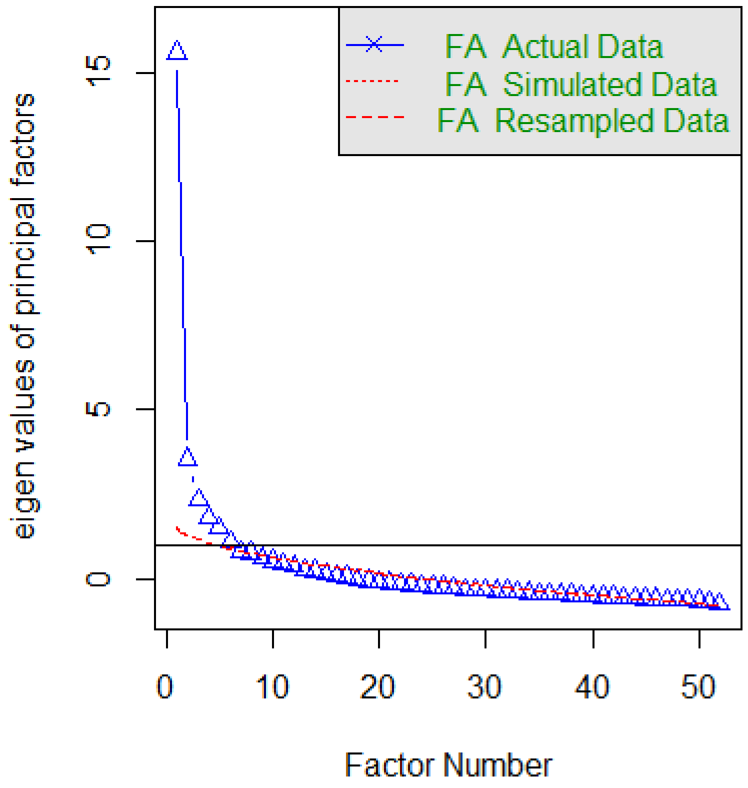

Supplementary Figure 1 Scree plot indicating that four factors would be suitable for the 50 Likert-type items of the BVQ.

Supplementary Table 2 Eigenvalues and percentages of variances and cumulative percentages for the 4 factors from the Exploratory Factor analysis.

	Total Variance Explained		
**Factor**	**Eigenvalue[a]**	**% Of Variance**	**Cumulative %**
1	7,35	35,00	35,00
2	2,72	12,97	47,97
3	2,30	10,95	58,91
4	1,28	6,10	65,01

[a]*Kaiser's criterion of eigenvalues, only eigenvalues greater than 1 are shown.*

Supplementary Table 3 Exploratory Factor Analysis with the items of the original constructs Oscillopsia (BVQO), Other physical symptoms (BVQP), Emotions (BVQE), Cognition (BVQC) and Imbalance (BVQI), of the Bilateral Vestibulopathy Questionnaire (BVQ).

**Item**	**Factor**				**Construct**
	**1**	**2**	**3**	**4**	
BVQO 8	.88				
BVQO 9	.77				
BVQO 10	.73				
BVQO 11	.70				Oscillopsia
BVQO 12	.77				
BVQP 13	.66				
BVQE 26		.79			
BVQE 28		.73			
BVQE 32		.62			Emotion
BVQE 33		.75			
BVQE 34		.71			
BVQC 19			.91		
BVQC 20			.86		Cognition
BVQC 21			.86		
BVQI 1				-.76	
BVQI 2				-.74	Imbalance
BVQI 3				-.68	
BVQI 4				-.73	
BVQI 7				-.80	
BVQC 16				-.80	

Notes: Extraction method; Principal Component Analysis; Rotation Method; direct Oblimin with Kaiser Normalization. Loadings larger than .60 are in shown.

Supplementary Table 4 Alterations of the 50 Likert type items of Bilateral Vestibulopathy Questionnaire after the Exploratory Factor Analysis (from van Stiphout et al. 2022 (1)). The items were developed in Dutch for the Dutch population. These items were translated into English for the purpose of this publication. Deleted items are marked in red.

	**Bilateral Vestibulopathy Questionnaire**	
	**Construct/Item**	**Answer scale**
	Imbalance	
1	I experience imbalance during daily activities.	*1 (never) – 6 (always)*
2	I experience imbalance when walking on uneven surfaces (like in the woods, at the beach or in the snow).	*1 (never) – 6 (always)*
3	I experience imbalance when walking in reduced light.	*1 (never) – 6 (always)*
4	When walking, I need to pay attention to the ground to avoid falling.	*1 (never) – 6 (always)*
5	I have fallen.	*1 (never) – 6 (always)*
6	I have come close to falling.	*1 (never) – 6 (always)*
7	I experience imbalance while changing positions (such as crouching, bending down, reaching or standing up).	*1 (never) – 6 (always)*
	Oscillopsia	
8	I have blurred vision while walking.	*1 (never) – 6 (always)*
9	I have blurred vision while travelling (such as being on a train, bus, car or on a bike).	*1 (never) – 6 (always)*
10	When walking, I have to stand still to recognize faces or to read (road) signs.	*1 (never) – 6 (always)*
11	I have blurred vision while chewing on my food.	*1 (never) – 6 (always)*
12	I have blurred vision when quickly turning my head.	*1 (never) – 6 (always)*
	Other psychical symptoms	
13	I experience difficulties with fast head movements, like turning my head to the right or left when crossing the street.	*1 (never) – 6 (always)*
14	I experience light-headedness when standing up fast.	*1 (never) – 6 (always)*
15	I feel tired.	*1 (never) – 6 (always)*
	Cognition	
16	I must pay close attention to my balance.	*1 (never) – 6 (always)*
17	I easily get lost in new places.	*1 (never) – 6 (always)*
18	I find it difficult to judge distances.	*1 (never) – 6 (always)*
19	I am forgetful.	*1 (never) – 6 (always)*
20	I find it difficult to concentrate.	*1 (never) – 6 (always)*
21	I experience difficulties with doing more than one thing at a time.	*1 (never) – 6 (always)*
22	I experience difficulties with doing things while walking.	*1 (never) – 6 (always)*
	Emotion	
23	I feel happy.	*1 (never) – 6 (always)*
24	I worry a lot.	*1 (never) – 6 (always)*
25	When performing daily activities, I feel confident.	*1 (never) – 6 (always)*
26	I feel lonely.	*1 (never) – 6 (always)*
27	I am afraid to fall.	*1 (never) – 6 (always)*
28	I feel I have control over my life.	*1 (never) – 6 (always)*
29	I am bothered by fast movements surrounding me.	*1 (never) – 6 (always)*
30	I am bothered by noisy environments.	*1 (never) – 6 (always)*
31	I am bothered by busy environments surrounding me.	*1 (never) – 6 (always)*
32	I don't feel confident when performing daily activities.	*1 (never) – 6 (always)*
33	I feel sad.	*1 (never) – 6 (always)*
34	I am embarrassed by my balance problems.	*1 (never) – 6 (always)*
	Behavior and limitations	
35	I feel <u>limited</u> in daily life activities.	*1 (never) – 6 (always)*
36	I <u>avoid</u> daily life activities.	*1 (never) – 6 (always)*
37	I feel <u>limited</u> when performing vigorous activities.	*1 (never) – 6 (always) – N/A*
38	I <u>avoid</u> performing vigorous activities.	*1 (never) – 6 (always) – N/A*
39	I feel <u>limited</u> when performing moderate activities.	*1 (never) – 6 (always)*
40	I <u>avoid</u> performing moderate activities.	*1 (never) – 6 (always)*
41	I feel <u>limited</u> in travelling.	*1 (never) – 6 (always)*

42	I avoid travelling.	*1 (never) – 6 (always)*
43	I feel limited when driving a car.	*1 (never) – 6 (always) – N/A*
44	I avoid driving a car.	*1 (never) – 6 (always) – N/A*
45	I feel limited in my social activities.	*1 (never) – 6 (always)*
46	I avoid social activities.	*1 (never) – 6 (always)*
47	I need to perform daily activities slower.	*1 (never) – 6 (always)*
	Social life	
48	My symptoms negatively affect my close relationships.	*1 (never) – 6 (always)*
49	People don't understand my problem.	*1 (never) – 6 (always)*
50	I need the help of other people to perform daily activities.	*1 (never) – 6 (always)*

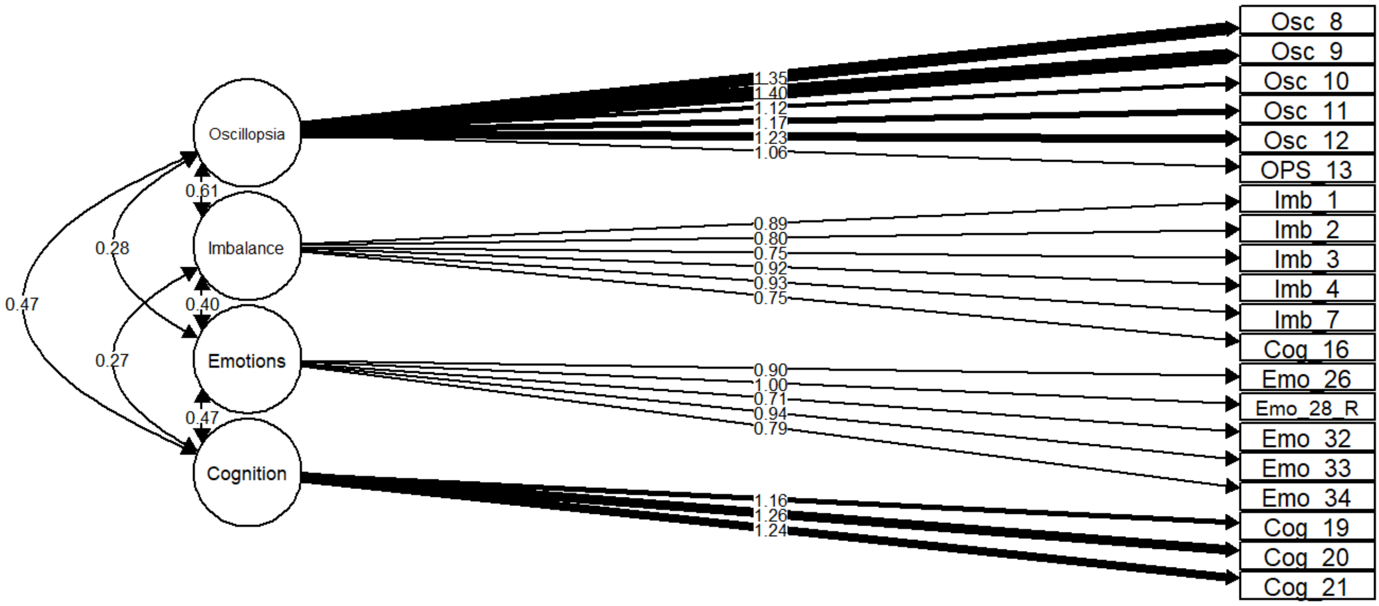

Supplementary Figure 2 Results of the confirmatory factor analysis: a visual representation of the structural equation of the 20-item model of the final version of the BVQ.

Supplementary Table 5 Mean total BVQ scores (standard deviation) for the four self-perceived dizziness related handicap groups (no handicap, mild handicap, moderate handicap, and severe handicap) according to the Dizziness Handicap Inventory (DHI) total score classification.

DHI classification	No handicap[a] (n=5)	Mild handicap[b] (n=24)	Moderate handicap[c] (n=45)	Severe handicap[d] (n=74)	ANOVA	
Mean BVQ (SD)	9.7 (4.1)	11.9 (2.3)	14.2 (2.1)	17.3 (2.3)	$F(3)=49.01$	$p<0.001$

[a]*Patient scores ranging from 0 to 15 points for the DHI total score were considered having no self-perceived dizziness related handicap.*
[b]*Patient scores ranging from 16 to 34 points for the DHI total score were considered having a mild self-perceived dizziness related handicap.*
[c] *Patient scores ranging from 36 to 52 points for the DHI total score were considered having a mild self-perceived dizziness related handicap.*
[d] *Patient scores equal to or higher than 54 points for the DHI total score were considered having a mild self-perceived dizziness related handicap.*

Supplementary Table 6. Results of the Bonferroni Procedure of Post-Hoc Analysis for comparing mean total BVQ scores with standard deviation (SD) for the four self-perceived dizziness related handicap groups (no handicap, mild handicap, moderate handicap, and severe handicap) according to the Dizziness Handicap Inventory (DHI) total score classification.

DHI_categories		Mean BVQ Total Score Difference	*p*-value
no handicap	mild handicap	-2,15	0,370
	moderate handicap	-4,51	0,000
	severe handicap	-7,61	0,000
mild handicap	moderate handicap	-2,36	0,001
	severe handicap	-5,46	0,000
moderate handicap	severe handicap	-3,10	0,000

References

1. van Stiphout L, Hossein I, Kimman M, Whitney SL, Ayiotis A, Strupp M, et al. Development and Content Validity of the Bilateral Vestibulopathy Questionnaire. Frontiers in Neurology. 2022;13.
